# Correlation Between Hip Strength, Foot Posture, and Lower Limb Dynamic Stability in Boxers

**DOI:** 10.7759/cureus.84934

**Published:** 2025-05-27

**Authors:** Geetha Sudha, Chandani Sharmila, Balaji Kuppusamy, Jibu George Varghese, Sai Aditya Raman, Thiagarajan K A

**Affiliations:** 1 Sports and Exercise Sciences, Sri Ramachandra Institute of Higher Education and Research, Chennai, IND; 2 Sports Physiotherapy, Sri Ramachandra Institute of Higher Education and Research, Chennai, IND; 3 Arthroscopy and Sports Medicine, Sri Ramachandra Institute of Higher Education and Research, Chennai, IND

**Keywords:** boxers, foot posture, hip strength, lower limb dynamic stability, y-balance test

## Abstract

Objective

This study aimed to assess lower limb components in boxers, focusing on the interplay between hip strength, foot posture, and lower limb dynamic stability. Given boxing's high-impact nature, understanding these factors is vital for performance optimization and injury prevention. Despite its significance, research in this area for boxers remains scarce.

Methodology

A cross-sectional design enlisted 60 professional boxers (45 male, 15 female) aged 18-35 years. Tests for hip strength, foot posture, and lower limb dynamic stability were conducted, with three trials for all tests, except for foot posture, which was assessed once. Data were recorded by the investigator, and the Pearson correlation test was used to analyze the relationships between hip strength, foot posture, and lower limb dynamic stability.

Results

The subjects averaged 20.17 ± 3.305 years, with mean heights, weights, and BMIs of 166.63 ± 10.810 cm, 74.93 ± 63.635 kg, and 26.87 ± 24.378, respectively. Significant correlations emerged between hip strength and lower limb dynamic stability in both limbs, while no significant differences were noted for other variables.

Conclusion

This study underscores the importance of evaluating foot and lower limb components in boxing. The strong correlation between hip strength and lower limb dynamic stability underscores the need to incorporate hip strength and foot posture assessments into boxer's training and injury prevention protocols.

## Introduction

Since 1904, the world-class and well-known combat sport of boxing has been included in every modern Olympic Games. Boxers are recognized for their agility, resilience, and physical strength. In addition to its popularity among athletes, boxing plays a significant role in contemporary sports culture [1]. When compared to other components of a punch (i.e., arms and trunk), boxers utilize dynamic and ballistic motion for punching performance [2]. Leg propulsion and foot positioning are crucial in boxing, as they facilitate the transfer of energy from the lower body to the upper limbs, thereby enhancing power generation. In a retrospective cohort study examining injuries during competition, 214 injuries were reported in 907 fights over 8.5 years among 545 professional boxers. This represents an injury rate of 24 per 100 fights [3]. Professional boxers must weigh themselves and reach their contest body mass 24 to 36 hours before the fight. A lower-level boxing match may last four rounds of three or two minutes each, with a one-minute break between rounds, whereas a top-level match may consist of 12 rounds of three minutes each, also with a one-minute break between rounds [4].

Hip strength is essential to boxing success, as it directly impacts punching force, balance, and overall body stability. During punching or defensive maneuvers, force is transferred from the lower limbs to the upper body through the hip joint, which serves as a vital link between the two. Deficiencies in hip strength can impair a boxer's ability to effectively generate and transfer force, leading to reduced punching power and an increased risk of injury [5].

Foot posture is equally important, as it significantly influences stability and overall movement efficiency. Proper foot alignment and arch support are crucial for absorbing impact forces and maintaining balance during sudden direction changes or pivoting motions. Boxers with poor foot posture may experience overuse injuries due to decreased stability, unbalanced weight distribution, and increased pressure on the lower limbs [6].

Maintaining stability is essential for equilibrium and control during dynamic movements, including pivoting, sudden direction changes, and rapid weight shifts. Dynamic stability plays a crucial role in maintaining optimal positioning, executing evasive maneuvers, and delivering powerful punches. Understanding the relationship between dynamic stability, foot posture, and hip strength provides valuable insights into the factors contributing to optimal performance and injury prevention [7].

Studies in other sports like taekwondo, kickboxing, and mixed martial arts (MMA) have also demonstrated a correlation between lower limb dynamic stability and hip strength. Strong hip muscles enhance joint stability, reduce the risk of lateral instability, and improve proprioception during rapid movements. However, limited research has specifically examined the relationship between hip strength and dynamic stability in boxing [8]. The effective transfer of power and energy through the lower limbs is fundamental to boxing techniques such as striking and footwork. Strong hip muscles facilitate the transfer of forces generated in the lower body, improving the stability of a boxer's punches and enhancing rapid footwork. Dynamic stability during these explosive movements is further optimized by the lower limb kinetic chain’s ability to transfer energy efficiently.

## Materials and methods

Sample population

The sample population consisted of elite male and female boxers aged between 18 and 35 years, with a minimum of two years of experience in the sport. Participants were excluded if they did not meet the inclusion criteria or had any of the following conditions: acute musculoskeletal injuries within the past seven days, systemic illnesses, comorbidities, neurological disorders, or a history of surgeries within the six months preceding the study.

Sample size

The study was conducted at the Centre for Sports Science (CSS), Sri Ramachandra Institute of Higher Education and Research (SRIHER). As there were no prior studies available to guide the sample size, the maximum number of athletes was recruited based on feasibility. All eligible athletes were included in the study between May 2023 and June 2023. A total sample of 60 athletes (n = 60) were included in the study.

Sample population recruitment

The researcher approached the boxing academy through the authorities of CSS after obtaining ethical clearance from the Institutional Ethics Committee at SRIHER. The investigator coordinated with the coaches of seven boxing academies and scheduled a meeting to provide a detailed explanation of the study. Both the athletes and their coaches were informed about the purpose and procedures of the study. Athletes who volunteered to participate provided written informed consent. Only athletes aged 18 years or older who met the inclusion criteria were included in the study. Participants were requested to complete a proforma detailing their demographic data, training history, and medical history.

Measurement tools

The measurement tools used in this study were a hand-held dynamometer and a Y-balance test kit.

Procedure

All measurements were collected by the examiner, following a testing sequence from non-fatiguing to fatiguing tests. Three trials were conducted for all tests except for foot posture assessment.

Hip strength assessment

Hip strength was measured using a hand-held dynamometer for the following movements: hip flexion, extension, abduction, adduction, internal rotation, and external rotation [9].

Hip Flexion

Participants were positioned in a supine position with the hip and knee flexed to 90 degrees and the ankle in dorsiflexion. The examiner placed the dynamometer at the proximal edge of the patella and applied resistance against hip flexion. Participants exerted maximum effort against the resistance. The standardized command given was: “Go ahead - push, push, push, push, and relax” (lasting 5 seconds).

Hip Extension

Participants lay in a prone position with the hip in a neutral position, the knee flexed to 90 degrees, and the ankle in dorsiflexion. The dynamometer was placed 5 cm proximal to the edge of the popliteal fossa, applying resistance against hip extension. Participants exerted maximum effort against the resistance. The standardized command given was: “Go ahead - push, push, push, push, and relax” (lasting 5 seconds).

Hip Abduction

Participants lay in a supine position with the hip in a neutral position. The dynamometer was placed 5 cm proximal to the lateral malleolus, applying resistance against hip abduction. Participants exerted maximum effort against the resistance. The standardized command given was: “Go ahead - push, push, push, push, and relax” (lasting 5 seconds).

Hip Adduction

Participants lay in a supine position with the hip in a neutral position. The dynamometer was placed 5 cm proximal to the medial malleolus, applying resistance against hip adduction. Participants exerted maximum effort against the resistance. The standardized command given was: “Go ahead - push, push, push, push, and relax” (lasting 5 seconds).

Hip Internal Rotation

Participants lay in a prone position with the hip in a neutral position and the knee flexed to 90 degrees. The dynamometer was placed 5 cm proximal to the lateral malleolus, applying resistance against hip internal rotation. Participants exerted maximum effort against the resistance. The standardized command given was: “Go ahead - push, push, push, push, and relax” (lasting 5 seconds).

Hip External Rotation

Participants lay in a prone position with the hip in a neutral position and the knee flexed to 90 degrees. The dynamometer was placed 5 cm proximal to the medial malleolus, applying resistance against hip external rotation. Participants exerted maximum effort against the resistance. The standardized command given was: “Go ahead - push, push, push, push, and relax” (lasting 5 seconds).

Outcome measures

Foot Posture Index

Participants stood with their feet shoulder-width apart. The foot posture index (FPI-6) was assessed through observation and palpation. Before assuming a relaxed stance with double limb support, participants were instructed to march in place for several steps. They were advised to avoid turning or adjusting their position during the examination, as this could influence foot posture. Participants remained still for approximately two minutes during the assessment [10].

Normalized Navicular Height Truncated

Participants were seated on a chair with their feet flat on the ground. The examiner identified the navicular tuberosity through palpation and marked it with a pen. Participants were then instructed to stand upright without shifting their weight or using their hands for support. The height of the mark from the floor was measured using a ruler, with the measurement recorded in millimeters. The test was repeated on the second foot, and the final value was presented as normalized navicular height truncated [11].

Y-Balance Lower Quadrant Test

The Y-balance test evaluates the dynamic stability of the lower extremities. Participants maintained a single-leg stance on the platform while reaching with the opposite leg in the anterior, posteromedial, and posterolateral directions. The test was repeated for both legs. The test was considered invalid and repeated if the participant failed to maintain a unilateral stance, touched the ground with the reaching foot, failed to return to the starting position without touching the ground, moved their hands from their hips, or pushed or kicked the indicator to artificially increase the reach distance. The maximum reach score for each direction was recorded across three trials. The composite score was calculated by summing the best of three trials in all three directions, dividing the total by three, and then normalizing the value based on limb length. Differences between the right and left leg were compared in all three directions to assess asymmetry. Limb length was measured from the anterior superior iliac spine (ASIS) to the medial malleolus using a measuring tape [12,13].

## Results

A total of 60 athletes were included in the study comprising 45 males and 15 females. The descriptive statistics for age, weight, and height are summarized below. The total number of subjects is 60, and the mean ± SD for age is 20.17 ± 3.305. The mean ± SD for height is 166.63 ± 10.810. The mean ± SD for weight is 74.93 ± 63.635. The mean ± SD for BMI is 26.87 ± 24.378.

Pearson correlation analysis in boxers showed varying relationships between foot posture, dynamic stability, and hip strength. The Y-balance test and FPI-6 had weak, nonsignificant correlations. However, moderate, significant negative correlations were observed between Y-balance and navicular drop, indicating that a lower Y-balance score was linked to a higher navicular drop (Tables 1, 2).

**Table 1:** Correlation analysis: Correlation matrix for right foot posture and lower limb dynamic stability in boxers * Correlation is significant at the 0.01 level (two-tailed).

	Foot posture index	Navicular drop	Y-balance test
Foot posture index	1		
Navicular drop	.340^*^	1	
Y-balance test	-.127	-.410*	1

**Table 2:** Correlation analysis: Correlation matrix for left foot posture and lower limb dynamic stability in boxers * Correlation is significant at the 0.01 level (two-tailed).

	Foot posture index	Navicular drop	Y-balance test
Foot posture index	1		
Navicular drop	.201	1	
Y-balance test	-.115	-.306*	1

Stronger correlations were found between Y-balance distances (anterior, posteromedial, and posterolateral) and hip strength, particularly in extension, adduction, and internal rotation. Additionally, composite Y-balance scores showed moderate positive correlations with various hip strength movements, notably extension and internal rotation (Tables 3-6).

**Table 3:** Correlation analysis: Correlation matrix for right side hip strength and lower limb dynamic stability in boxers * Correlation is significant at the 0.01 level (two-tailed).

	Flexion	Extension	Abduction	Adduction	External rotation	Internal rotation	Y-balance test
Flexion	1						
Extension	.224	1					
Abduction	.496*	-.017	1				
Adduction	.562*	.143	.557*	1			
External rotation	.504*	.134	.778*	.578*	1		
Internal rotation	.020	.026	.338*	.155	.146	1	
Y-balance test	.115	.282^*^	.195	.304^*^	.141	.428^*^	1

**Table 4:** Correlation analysis: Correlation matrix for left side hip strength and lower limb dynamic stability in boxers * Correlation is significant at the 0.01 level (two-tailed).

	Flexion left	Extension left	Abduction left	Adduction left	External rotation left	Internal rotation left	Y-balance test left
Flexion left	1						
Extension left	.633*	1					
Abduction left	.333*	.242	1				
Adduction left	.691*	.556*	.255*	1			
External rotation left	.228*	.191	.404*	.244	1		
Internal rotation left	.544*	.319*	.289*	.437*	.259*	1	
Y-balance test left	.195	.162	.110	.141	.306*	.347*	1

**Table 5:** Hip strength and Y-balance test - right side

	Male				Female			
	Mean	±SD	Min	Max	Mean	±SD	Min	Max
Hip strength								
Flexion	207.81	44.528	101.67	292.55	153.74	32.346	90.55	201.04
Extension	231.53	118.005	100.27	897.33	184.17	34.622	115.39	239.93
Abduction	160.26	39.467	75.53	235.03	144.12	27.239	94.47	185.35
Adduction	113.68	29.949	39.88	170.64	95.96	28.081	53.61	145.14
External rotation	111.36	27.400	61.46	174.23	96.23	30.398	49.36	159.52
Internal rotation	101.69	40.009	41.52	230.08	91.01	55.228	43.80	235.36
Y-balance test								
Anterior	56.45	45.000	42.00	74.00	52.30	3.506	44.60	57.00
Posteromedial	77.38	10.720	57.30	104.60	73.04	7.699	60.60	84.60
Posterolateral	79.21	10.817	57.30	107.30	72.52	6.985	62.30	89.30

**Table 6:** Hip strength and Y-balance test - left side

		Male				Female		
	Mean	±SD	Min	Max	Mean	±SD	Min	Max
*Hip strength*								
Flexion	189.76	32.817	123.89	255.95	146.07	21.562	98.07	178.66
Extension	198.07	50.426	90.13	307.93	174.32	34.815	107.55	236.01
Abduction	154.15	57.433	86.53	481.74	133.69	26.046	85.97	178.48
Adduction	117.83	24.586	62.11	169.98	99.88	28.815	53.02	156.58
External rotation	113.36	224.621	61.13	180.77	107.43	62.162	50.34	311.59
Internal rotation	98.97	28.214	27.13	146.77	82.23	22.202	55.25	131.46
*Y-balance test*								
Anterior	54.97	7.547	41.00	78.30	52.38	5.090	44.60	60.30
Posteromedial	76.76	12.493	53.80	105.60	74.81	8.452	60.30	92.30
Posterolateral	76.71	11.088	55.30	110.00	74.11	5.912	67.00	90.30

This is one of the rare studies, to the best of our knowledge, to assess and compare selected lower limb components in boxers. The findings indicate a statistically significant difference in more than one of the tested parameters. This highlights the importance of not only focusing on upper limb components and skills but also considering the lower limb, which plays a crucial role in maintaining the stability and strength required for the sport.

## Discussion

In this study, subjects were boxers with different levels of play (state and national). The study indicates a positive correlation between hip adduction and internal rotation in anterior, posteromedial, and posterolateral reach on both the right and left sides. This is due to boxing stances, where the hips adduct (move toward the center line of the body) and internally rotate (rotate inwardly toward the midline of the body) to maximize stability and generate force. These movements call for bringing the leg closer to the center line of the body. The raising of the thigh and flexion of the hip joint is accomplished by the hip flexor muscles, which include the iliopsoas and rectus femoris. When doing boxing moves like knee lifts and front kicks, these muscles are actively used. Boxers who have strong hip flexors can lift their legs higher during these movements, which may help them keep their balance during the anterior reach portion of the Y-balance test. When performing actions like throwing punches and maintaining a powerful stance, the hip extensor muscles, especially the gluteus maximus, and hamstrings, are essential for producing power and stabilizing the hip joint. These muscles help govern the descent of the leg back to the ground and are crucial for producing force through the hips during reaching actions.

In order to perform well, a boxer needs strong hips. Powerful punches, the transmission of force from the lower to the upper body, and maintaining stability while moving all depend on a strong hip. Boxers with stronger hips produce punches with more force, move more quickly, and perform better overall in the ring. It describes the force generated from the rear foot with rotation of hip to shoulder to the weight transmitted to the lead foot. Boxing stance techniques involve two positions a fighter can take, which are the typical orthodox stance, in which the left (lead) foot is in the front and the right (rear) foot is positioned in the back; and the southpaw stance, in which the right (rear) foot is in the front, the left (lead) foot is positioned in the back.

Although this is not always the case, southpaws are typically left-handed. However, it all depends on the fighter's natural posture. This stance position enables a boxer to effectively move in the ring and to both attack and defend while constantly remaining in a balanced position [14]. It determines that both the feet and leg play an important role in stability and balance in boxers. Foot posture describes how the feet are aligned and placed during static and dynamic movements. Maintaining proper foot posture is essential for preserving balance, preventing injuries like ankle sprains, and maintaining the stability of the lower limbs. In order to move quickly and change directions quickly, boxers significantly rely on the stability of their lower limbs. A boxer with good foot posture has a lesser chance of losing balance. A better ankle proprioception can be a good supportive factor for this. Weak hip muscles might result in different lower limb mechanics, which can change how the feet are positioned and how a boxer moves. For instance, insufficient hip strength might result in excessive leg inward or outward rotation, which can produce aberrant pronation or supination of the feet.

The present study identified varied relationships among foot posture, lower limb dynamic stability, and hip strength in professional boxers. Descriptive analysis indicated that the cohort comprised 60 athletes (45 males and 15 females) with a mean age of 20.17 ± 3.305 years, height of 166.63 ± 10.810 cm, weight of 74.93 ± 63.635 kg, and BMI of 26.87 ± 24.378. Correlation analysis revealed that the association between foot posture, as measured by the FPI, and dynamic stability using the Y-balance test was weak and statistically nonsignificant. In contrast, a moderate and statistically significant negative correlation was found between Y-balance scores and navicular drop, suggesting that increased navicular drop was associated with reduced dynamic balance performance. Furthermore, notable positive correlations were observed between hip muscle strength, particularly in extension, adduction, and internal rotation, and reach distances in the Y-balance test (anterior, posteromedial, and posterolateral directions). The composite Y-balance scores also exhibited moderate positive correlations with various hip strength parameters, indicating the relevance of proximal muscle function in maintaining lower limb dynamic stability.

Boxers rely heavily on their hip strength and foot posture for stability and performance. Strong hip muscles, especially the flexors and extensors, are essential for generating power in punches and maintaining balance. Skilled boxers utilize their legs more efficiently, contributing significantly to punch force [15]. Foot posture plays a crucial role in maintaining stability and preventing injuries like ankle sprains. Proper foot alignment enhances dynamic stability during fast movements in the ring [16]. Weak hips or poor foot posture can lead to biomechanical issues and increase the risk of injury. Training focusing on hip strength and foot posture can improve performance and reduce injury likelihood [17]. Different foot positions, like overpronation or supination, affect joint stability and movement efficiency [18]. Dynamic stability is crucial in boxing due to the need for quick, unpredictable movements [19]. Understanding the interaction between hip strength, foot posture, and dynamic stability requires further investigation [20]. Kinematic and kinetic factors, such as hip flexion and quadriceps strength, are key predictors of dynamic balance in boxers [21]. Maintaining lower arch height enhances equilibrium by improving foot-ground contact and proprioception.

Limitations of the study

This study has certain limitations, including a limited sample of female boxers, a lack of representation from players across different states, and the inclusion of participants exclusively trained in the orthodox stance.

Future recommendations of the study

Future research should explore the correlation between hip strength, foot posture, and lower limb dynamic stability in southpaw stance boxers and compare these variables between orthodox and southpaw stances. Coaches and trainers should integrate targeted exercises for hip strengthening, foot alignment, and dynamic stability into training programs to enhance performance and reduce injury risk. Longitudinal studies are recommended to assess the long-term impact of training interventions on lower limb stability. Additionally, future research should investigate other contributing factors, such as core strength, proprioception, and ankle range of motion, to gain a comprehensive understanding of lower limb stability in boxers.

## Conclusions

The study reveals a positive correlation between hip strength and lower limb dynamic stability in boxers. Stronger hip muscles contribute to better balance and control during dynamic movements, which are essential for enhancing ring performance and reducing the risk of lower limb injuries. Additionally, the findings indicate no correlation between foot posture and lower limb dynamic stability in boxers. Factors such as hip strength, core stability, and overall training may have a greater impact on lower limb stability than foot posture alone. Boxing involves a wide range of complex movements that require the coordination of multiple muscle groups, and foot alignment may be just one of several contributing factors.
